# Biodegradation of flubendiamide by a newly isolated *Chryseobacterium* sp. strain SSJ1

**DOI:** 10.1007/s13205-015-0347-9

**Published:** 2016-01-14

**Authors:** Shrinivas S. Jadhav, M. David

**Affiliations:** Environmental and Molecular Toxicology Laboratory, Department of PG Studies in Zoology, Karnatak University, Dharwad, Karnataka 580003 India

**Keywords:** Flubendiamide, Phthalic acid diamide, Biodegradation, *Chryseobacterium indologenes*

## Abstract

Flubendiamide, as a new class (Phthalic acid diamide) of pesticide with a wide spectrum of activity against lepidopteran pests extensively used alone or in combination with other insecticides in agriculture system to get protection from insect pests. Due to high specificity and limited approach towards non-target organism, the extensive use of this pesticide as an alternate for organophosphate and organochlorine pesticides, causing an eventual increase in environmental pollution. Five flubendiamide-resistant bacterial strains were isolated during the present study from agriculture soil considering previous history of pesticide application. Minimal inhibitory concentration of all the isolates showed strain SSJ1 was most efficient flubendiamide resistant organism. Biochemical tests and molecular sequencing of 16s rRNA was carried out which confirmed the isolate as *Chryseobacterium indologenes* strain SSJ1. UV–visible spectrophotometer study revealed that 89.06 % initial pesticide was removed by the isolate at optimum temperature of 35 °C and pH 7.0 with 5 days incubation period and is further confirmed by high-performance liquid chromatography (HPLC) analysis. Results of the present study however, suggest strain SSJ1 is most resistant to flubendiamide and can possibly be applied in the bioremediation of flubendiamide contaminated soils.

## Introduction

Pesticides pose wide variety of disturbances in aquatic as well as terrestrial organisms, which include morphological, behavioural or physiological disturbances (Xing et al. 2012; Mansour and Mossa 2011; David et al. 2012). These xenobiotics, however, enter food chain and gain access into humans through biomagnifications (Ellgehausen et al. 1980) thereby affecting physiological activities. Indiscriminate use of these pesticides renders a greater threat to living organisms as well as environmental sustainability. Many studies were undertaken which confirm the neurological, physiological disturbances due to pesticide toxicity (David et al. 2015).

Recent years, formulating a new class of chemical with a potential for acting on specific pest posing no harm to non-targeted organism was of prime importance. One such Phthalic acid diamide class of pesticide flubendiamide (C_23_H_22_F_7_IN_2_O_4_S) was reported to be effective against a broad spectrum of lepidopteran insect pest with a relatively low toxicity to non-targeted organisms (Sarkar et al. 2014). This formulation works by disrupting ion channels and thereby paralysing the muscle fibres of the gut (Nishimatsu et al. 2005), therefore, the larvae stops feeding and eventually dies from starvation (Ebbinghaus-Kintscher et al. 2006). Flubendiamide is been widely used in Indian agriculture system for cultivation of rice and cotton, to get protection from pests (Government of India, Ministry of Agriculture, Department of Agriculture and Cooperation 2009). Apart from these two crops, this pesticide is also been used in the cultivation of fruiting vegetables, stone fruit, corn, cotton, grape, okra, tobacco, etc. (EPA 2010).

Even though precisely targeted formulations of flubendiamide are expected to be safe for non-target organisms, yet, several recent studies have shown toxic potentials of flubendiamide on many non-target organisms. Neurotoxic potentials and morphological anomalies in *drosophila melanogaster* have been reported (Sarkar et al. 2014). Acute and joint toxicity of flubendiamide was reported by Wei et al. (2014) on Chinese tiger frog *Hoplobatrachus chinensis* tadpoles. Alterations in protein metabolism of fresh water fish *Labeo rohita* have been reported by Nirmalakallagadda and Rathnamma (2014). Disruption of enzyme activity in tropical soil after flubendiamide application was reported (Shrinivas and David 2015). These findings not only provide enough scientific evidences for flubendiamide toxicity to non-target organisms, and also is undesirable with ecological perspective, and furthermore, its fate as serious environmental pollutant.

Present study aims to explore the available means of detoxifying this pesticide using possible ways. One such economic and eco-friendly way of achieving this is by bioremediation using native microorganisms. Bioremediation is a process of cleaning up of contaminated soils using biological means. This process ultimately aims towards forcing these native microbial populations to use contaminant (pesticide) as a source of carbon and nutrient for their growth and development (Islas-garcia et al. 2015). Furthermore, it is necessary to perform physicochemical characterisation of contaminated soil in order to find out whether the native microbial species is capable of degrading the pesticide by biostimulation. However, unfortunately, there is no literature available so far for the bioremediation of flubendiamide in submerged culture by native bacteria. Hence, the present work was carried out in order to attempt isolation of flubendiamide resistant bacteria from the contaminated soil and to evaluate the potentials of these isolates in degrading the pesticide. These bacterial isolates could then be used in future to ward off or to limit the concentration of flubendiamide in agriculture systems thereby preventing them from reaching non-target organisms.

## Materials and methods

### Chemicals and media

Flubendiamide (98 % pure) was procured from TATA Rallis Co India and other molecular biology reagents were bought from Sigma-Aldrich. All other chemicals and reagent used in present study were procured from Himedia and were of analytical or HPLC grade.

The mineral salts (MS) media, pH 7.0, contained NH_4_NO_3_ (1.0 g/L), K_2_HPO_4_ (1.5 g/L), KH_2_PO_4_ (0.5 g/L), NaCl (1.0 g/L), MgSO_4_·7H_2_O (0.1 g/L), and FeSO_4_ (0.025 g/L). The Nutrient agar (NA) medium, pH 7.0, contained yeast extract 5 g/L, tryptone 10 g/L, and NaCl 10 g/L.

### Sample collection and physicochemical analysis

Soil samples were collected from the three different sites in groundnut cultivating soil of Dharwad district, Karnataka, India, with a previous history of flubendiamide use. Soil samples were collected from 10 to 20 cm depth, aseptically put into labelled 250 mL sterile culture bottles and taken to laboratory in ice-box for characterisation and isolation of flubendiamide resistant bacteria.

Mineral matter of soil samples such as sand, silt, and clay contents were analysed with use of different sizes of sieves by following the method of Alexander (1961). Soil pH was measured at 1:1.25 soils to water ratio in systronics digital pH metre with calomel glass electrode assembly. Organic carbon content in soil samples was estimated by the Walkley and Black method and the organic matter was calculated by multiplying the values with 1.72 (Jackson 1971). Electrical conductivity of soil samples after addition of 100 ml distilled water to 1 g soil samples was measured by conductivity bridge. Total nitrogen content in soil samples was determined by the method of micro-Kjeldhal method (Jackson 1971). Content of inorganic ammonium-nitrogen in soil samples was determined by Nesslerization method (Jackson 1971) and contents of nitrite-nitrogen (Barnes and Folkard 1951) and contents of nitrate–nitrogen by Brucine method (Ranney and Bartlett 1972) after extraction with water were determined, respectively.

### Enrichment and isolation of flubendiamide-resistant bacteria

Microorganisms were extracted from 10 g of soil samples with 90 mL sterile distilled water, phosphate buffer 50 mM, pH 7, or peptone water in shaker (60 min, 35 °C and 120 rpm). Serial dilutions of soil suspension were made up in 10 mL sterile distilled water and 0.1 mL was taken from 10^−5^ and 10^−6^ dilution, and was then plated on Petri dishes containing NA. Based on colony morphology, distinct colonies were picked and streaked over MS agar media containing flubendiamide as a sole carbon and nitrogen source. This was incubated at 28–32 ^°^C for 4 days. These colonies were sub cultured periodically on pesticide supplemented medium until pure colonies were obtained. The pure cultures were picked out and streaked on to nutrient agar tubes and frozen in glycerol for conservation.

### Minimum inhibitory concentration

Minimum inhibitory concentration (MIC) of flubendiamide was determined for the isolated bacterial strains by inoculating 1 mL of bacterial culture (OD_600_ = 0.4) in 100 mL of MS media supplemented with varying concentration of flubendiamide. These flasks were incubated at 35 °C on a rotary shaker for 48 h. After incubation, 100 µL of the sample was plated on nutrient agar and incubated at 35 °C for bacterial growth. Colony forming units (cfu) were then counted and the strain showing maximum tolerance to flubendiamide was selected for further studies.

### Characterisation of efficient strain

Most resistant isolates from MIC was selected for further studies. Identification and characterisation of these strain was done by standard biochemical techniques (Hort et al. 1994). 16S rRNA gene sequencing was performed to confirm the isolate strain. Isolation and purification of genomic DNA were carried out according to InstaGene™ Matrix Genomic DNA isolation kit Catalogue # 732-6030. 16S rRNA gene was amplified using the 27F primer (5′-AGAGTTTGATCMTGGCTCAG-3′) and 1492R primer (5′-TACGGYTACCTTGTTACGACTT-3′). The PCR amplification was carried out using 1 µL template DNA in 20 µL of PCR reaction solution. The amplification reaction was cycled as follows: Initial denaturation at 94 °C for 5 min and denaturation at 94 °C for 45s, annealing at 55 °C for 60s, extension at 72 °C for 60s and final extension at 72 °C for 15 min for 35 amplification cycles. Purification of PCR products were done by removing unincorporated PCR primers and dNTPs from PCR products by using Montage PCR Clean up kit (Millipore). The product was directly sequenced with the primer using Genetic Analyzer (Yaazh Xenomics Pvt Ltd, Chennai, India).

### Growth kinetics and degradation study

To determine growth of bacterial isolate, 0.1 mL of overnight culture was inoculated in 100 mL of MSM and nutrient broth with 100 mg/L concentration of flubendiamide. Flasks without pesticide were maintained as controls. Both test and control samples were incubated at 35 ± 2 °C on rotary shaker at 100 rpm. Optical density was measured at regular intervals of time up to 5 days of incubation at 600 nm.

Biodegradation of flubendiamide was studied in submerged culture of MSM flasks inoculated with 1.0 mL of 24 h old bacterial culture. Initial pesticide load was maintained at concentration of 100 mg/L and incubated at different temperature and pH range. 10 mL fraction was taken out at each time interval and centrifuged at 5000 rpm for 10 min. The supernatant was collected and absorbance was measured at 230 nm by UV–visible spectrophotometer (Secomam Anthelie Advanced, Model V2.5b) and then by HPLC. Quantification was made against standard graph plotted with 98 % pure flubendiamide. Amount of flubendiamide in media after incubation was estimated and percent degradation was calculated by using the formula.

$$\frac{{C_{\text{o}} - C_{\text{t}} }}{{C_{\text{o}} }} \times 100,$$

where *C*_o_ is initial concentration and *C*_t_ concentration at time ‘*t*’ (Vijayalakshmi and Usha 2012).

Samples were prepared for HPLC by solvent extraction method and analysed using ODS2 C18 reversed phase column and UV–Vis detector at 230 nm. HPLC grade acetonitrile and water were used as mobile phase (60:40). The flow rate was maintained at 1 ml/min and sample injection volume of 20 µl (Paramasivam and Banerjee 2012). All experiments were carried out in triplicates and results are taken as mean value for each set.

## Results and discussion

In the present study, an attempt was made to isolate flubendiamide resistant bacteria from contaminated agriculture soils of Dharwad district to study their pesticide degrading capabilities. The soil samples were isolated from sites which had previous history of pesticide application and would add to the isolation of strains with desired resistance towards flubendiamide. Physicochemical analysis of soil showed it was nutrient rich with high organic matter (Table 1). This would imply there is no need for further nutrient fortification for bacterial growth. The soil sample was enriched with flubendiamide in MS media and five bacterial strains were isolated. These strains were named as SSJ1, SSJ2, SSJ3, SSJ4 and SSJ5, respectively. Similar study was carried out by Jayanthi and Srujana (2014) successfully isolated four malathion degrading bacteria out of which, strain *Achromobacter xylosoxidans* utilised malathion as sole carbon and nitrogen source and could effectively reduce the pesticide concentration to undetectable level within 5 days.

**Table 1:** Physicochemical property of the soil

Parameter	
Sand (%)	66.0
Silt (%)	24.2
Clay (%)	12.6
pH^a^	7.3
Water holding capacity (ml g^−1^ soil)	0.61
Electrical conductivity (m mhos)	230
Organic matter (%)^b^	1.44
Total nitrogen (%)^c^	0.077
NH_4_^+^—N (µg g^−1^ soil)^d^	7.56
NO_2_^−^—N (µg g^−1^ soil)^e^	0.39
NO_3_^−^—N (µg g^−1^ soil)^f^	0.85

^a^1:1.25 = Soil:Water slurry

^b^Walkley–Black method (Jackson 1971)

^c^Micro-Kjeldhal method (Jackson 1971)

^d^Nesslerization method (Jackson 1971)

^e^Diazotization method (Barnes and Folkard 1951)

^f^Brucine method (Ranney and Bartlett 1972)

The MIC of flubendiamide was studied for each of the bacterial isolate. Among five isolates, SSJ1 exhibited a maximum tolerance of up to 1000 mg/L. This strain was further selected and processed for the degradation studies of flubendiamide. Bacterial isolate SSJ1 was found to be yellow-pigmented Gram negative, non-motile rod. The freshly grown cultures showed positive results for indole production, oxidase, catalase, urease, protease while negative for nitrate reduction, glucose acidification, maltose assimilation and thereby presumably identified as *Chryseobacterium* sp. Since, identification of the isolate by biochemical tests lead to unclear results, molecular characterisation was carried out by partial sequencing of 16s rRNA gene. The homology of a partial sequence with that of from NCBI database was compared and confirmed the identity of a strain as *Chryseobacterium indologenes* and it was designated and submitted to genbank as *C.* *indologenes* strain SSJ1 with the accession number KP406152.

**Fig. 1:**
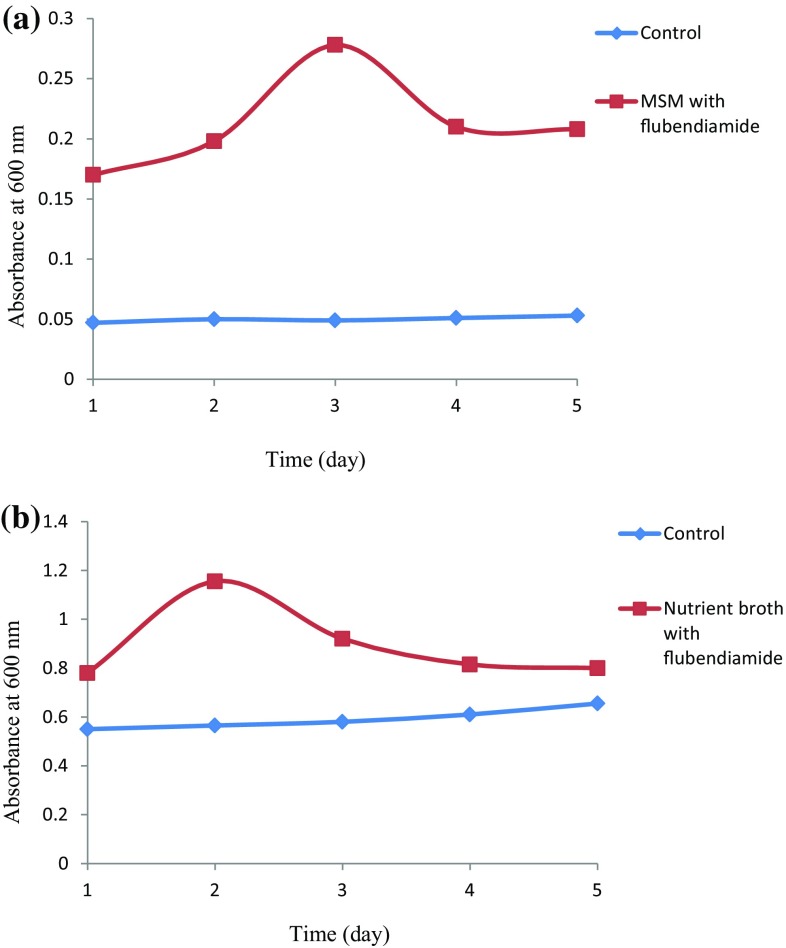
Growth pattern of *Chryseobacterium indologenes* strain SSJ1 in MSM (**a**), Nutrient broth (**b**), in presence and absence of flubendiamide

Growth kinetics and degradation profile was studies successively following the characterisation of efficient flubendiamide strain. MS media and nutrient broth were used to study the growth kinetics of the strain SSJ1. Media was inoculated with strain SSJ1 and amended with 100 mg/L flubendiamide concentration, and the isolate utilised the flubendiamide as a sole carbon and nitrogen source (Fig. 1a). Growth was also observed in nutrient broth which showcased an elevated growth pattern in presence of necessary nutrients (Fig. 1b). Enhancement in the culture growth was observed when flubendiamide was an additional carbon source in nutrient broth. This indicates presence of additional carbon source could be a counter selective to the organism that used flubendiamide for its growth. Our study is in agreement with Jayanthi and Srujana (2014) who studied degradation capability of *A. xylosoxidans* strain JAS11 in degrading 1000 mg/L malathion in MSM and nutrient broth. Similar study was carried out by Bhalerao and Puranik (2009) with fungal isolate *Aspergillus oryzae* for the degradation of monocrotophos.

**Fig. 2:**
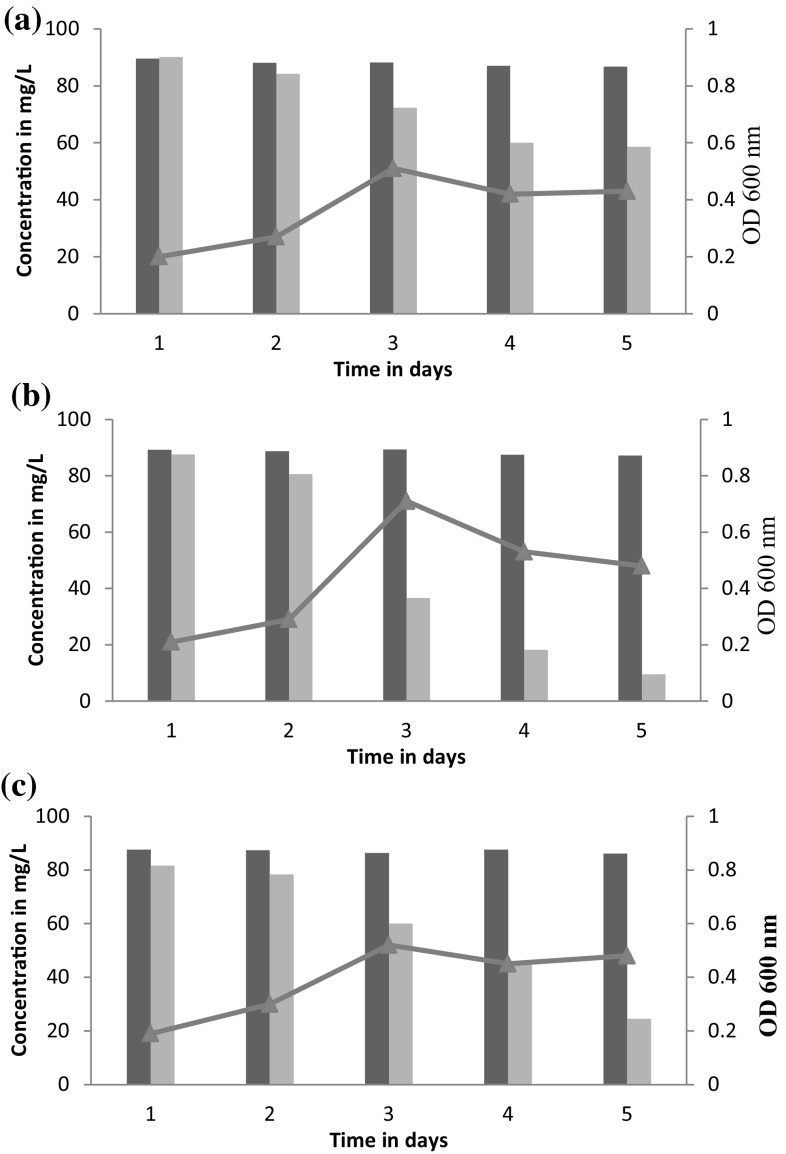
Biodegradation pattern of flubendiamide in submerged cultures. *Light coloured bars* show flubendiamide concentration, *dark coloured bars* indicate control (without inoculation), and *line* shows biomass. **a** 30 °C, pH 5.0; **b** 35 °C, pH 7.0; **c** 40 °C, pH 8.5

**Fig. 3:**
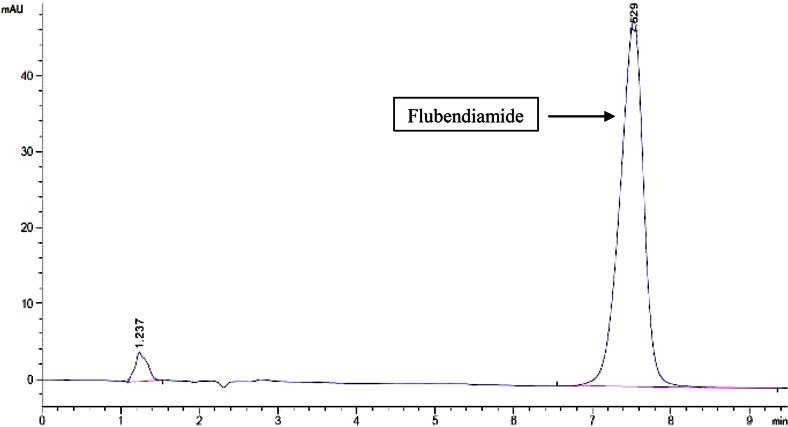
HPLC chromatogram of standard flubendiamide

During field application of the pesticide, the environmental conditions with respect to pH and temperature vary greatly and are almost impossible to attain a defined state. The degradation potential of the strain SSJ1 therefore was studied in culture media with varying physical parameters such as pH and temperature. At pH 5.0 and temperature 30 ± 2 °C, pesticide scavenging ability of strain SSJ1 followed steady path of decrease of 9.9, 15.79, 27.7, 40.0 to 41.4 % at day 1, 2, 3, 4 and 5 respectively (Fig. 2a). Temperature and pH of 35 °C and 7.0 are considered ideal for optimal growth of bacteria. Concentration of flubendiamide exhibited rapid decent of 52 % on day 3 and 79 % on day 4 as compared to control. Further, at day 5, the initial concentration went from 100 to 9.5 mg/L which accounts for the removal of about 89.1 % of the initial pesticide concentration (Fig. 2b). Further, it was confirmed by HPLC analysis with flubendiamide standards (Fig. 3). The fifth day sample showed little or no peak, and this could be interpreted as analytical evidence for the bio removal of flubendiamide.

However, increase in temperature up to 40 ± 2 °C and high basicity followed almost similar trend as with neutral pH and 35 °C with about 24.4 mg/L pesticide at day 5 which almost 71 % decrease with control from initial load (Fig. 2c). Temperature and pH had a significant effect on degradation of flubendiamide. It was observed that neutral pH and optimum temperature of 35 °C contributed more in bringing down the pesticide concentration whereas, more acidic and more basic conditions hindered the growth of bacteria and thus reduced their efficiency in scavenging the pesticide. Similar effect was observed with respect to temperatures at 30 and 40 °C.

## Conclusion

The development and use of different formulations of pesticides in control of pests of agriculture importance have a nugatory effect on environmental sustainability and majority of these mainly reach non-target sites thus entering into food chain as well. Repeated applications of these pesticides have lead to the inception of resistant microorganisms and these microbes both bacteria as well as fungi can be isolated from these contaminated fields. In this work, flubendiamide resistant bacteria were isolated and tested for their degrading capabilities. Among five effective isolates, strain SSJ1, identified as *C. indologenes* showed maximum resistance of up to 1000 mg/L flubendiamide and degraded about 89.06 % of initial pesticide in 5 days incubation. HPLC reading are confirmative for this removal of pesticide as there was no peak elevation when compared with standards. Further studies are needed to assess effect of consortia on degradation, as well as some research on possible gene responsible for the degradation.
